# Metalloproteinases 1 and 3 as Potential Biomarkers in Breast Cancer Development

**DOI:** 10.3390/ijms22169012

**Published:** 2021-08-20

**Authors:** Angela Ximena Argote Camacho, Amanda Rocío González Ramírez, Alejandro José Pérez Alonso, Juan David Rejón García, María Auxiliadora Olivares Urbano, Pablo Torné Poyatos, Sandra Ríos Arrabal, María Isabel Núñez

**Affiliations:** 1 Department of Surgery, Clínico San Cecilio University Hospital, 18012 Granada, Spain; angelitaxa29@gmail.com; 2 Bio-Health Research Foundation of Eastern Andalusia—Alejandro Otero (FIBAO), 18012 Granada, Spain; agonzalez@fibao.es; 3 Department of Surgery, Virgen de las Nieves University Hospital, 18014 Granada, Spain; apma85@hotmail.com; 4 Andalusian Tumour Bank Network, 18012 Granada, Spain; jdrg@juntadeandalucia.es; 5 Department of Radiology and Physical Medicine, University of Granada, 18012 Granada, Spain; auxiou@ugr.es; 6 Department of Surgery and Its Specialties, University of Granada, 18012 Granada, Spain; ptorne@ugr.es; 7 Institute of Biopathology and Regenerative Medicine (IBIMER), University of Granada, 18016 Granada, Spain; 8 Biosanitary Research Institute, ibs.Granada, 18012 Granada, Spain

**Keywords:** breast cancer, metalloproteinases, immunohistochemical expression, epithelial-to-mesenchymal transition (EMT), biomarkers, diagnostic factors, extracellular matrix, MMPs, MMP inhibitors

## Abstract

Breast cancer continues to be one of the main causes of morbidity and mortality globally and was the leading cause of cancer death in women in Spain in 2020. Early diagnosis is one of the most effective methods to lower the incidence and mortality rates of breast cancer. The human metalloproteinases (MMP) mainly function as proteolytic enzymes degrading the extracellular matrix and plays important roles in most steps of breast tumorigenesis. This retrospective cohort study shows the immunohistochemical expression levels of MMP-1, MMP-2, MMP-3, and MMP-9 in 154 women with breast cancer and 42 women without tumor disease. The samples of breast tissue are assessed using several tissue matrices (TMA). The percentages of staining (≤50%–>50%) and intensity levels of staining (weak, moderate, or intense) are considered. The immunohistochemical expression of the MMP-1-intensity (*p* = 0.043) and MMP-3 percentage (*p* = 0.018) and intensity, (*p* = 0.025) present statistically significant associations with the variable group (control–case); therefore, expression in the tumor tissue samples of these MMPs may be related to the development of breast cancer. The relationships between these MMPs and some clinicopathological factors in breast cancer are also evaluated but no correlation is found. These results suggest the use of MMP-1 and MMP-3 as potential biomarkers of breast cancer diagnosis.

## 1. Introduction

There were an estimated 19.3 million new cases of cancer (18.1 million excluding non-melanoma skin cancer) and almost 10.0 million deaths from cancer (9.9 million excluding non-melanoma skin cancer) worldwide in 2020 [1], with the most commonly diagnosed cancers worldwide being female breast cancer (11.7%), followed by lung (11.4%), colorectal (10.0%), prostate (7.3%), and stomach (5.6%) cancers. Lung cancer remained the leading cause of cancer death, with an estimated 1.8 million deaths (18%), followed by colorectal (9.4%), liver (8.3%), stomach (7.7%), and female breast (6.9%) cancers; however, the COVID-19 pandemic is known to have affected the number of cancer diagnoses in many countries, so the actual number of cancers diagnosed in 2020 will likely have been lower. Global estimates also indicate that the number of new cases will increase in the next two decades to 30.2 million new cases per year by 2040 [2]. The overall impact of COVID-19 on cancer deaths due to delays in diagnosis has been reported [3,4]. Particularly, the use of immune checkpoint inhibitors on cancer management has been analyzed by some authors [5].

Female breast cancer (BC) has now surpassed lung cancer as the leading cause of global cancer incidence in 2020, with an estimated 2.3 million new cases, representing 11.7% of all cancer cases. It is the fifth leading cause of cancer mortality worldwide, with 685,000 deaths. Among women, breast cancer accounts for 1 in 4 cancer cases and for 1 in 6 cancer deaths, ranking first for incidence in most countries [2]. In 2021, BC will be the most frequent tumor diagnosed in women in Spain, with a total of 33,375 new cases [6].

BC is the fourth leading cause of cancer death in Spain in both sexes, but the first cause of death in women in Spain in 2020 due to cancer. The mortality rate of this pathology is lower than the incidence, due to its high prevalence; Of the 32,953 patients diagnosed with breast cancer in 2020 in Spain, only 5.8% died (1911), so its prevalence at 5 years is 144,233 women [2,3].

Long years of research have demonstrated the fundamental role played by proteases in embryonic development, in the repair and remodeling of tissues, as well as in the processes of invasion by malignant cells that lead to infiltration and metastasis, properties that they influence the malignancy of cancer [7,8].

Metalloproteinases (MMP) are a family of zinc-dependent endopeptidases secreted by tumor and stromal cells, which participate in the degradation of the extracellular matrix (ECM) and the barriers of the basement membrane [9]. Their activity is regulated by specific inhibitors known as tissue inhibitors of metalloproteinases, called TIMPs [10]. Currently, 24 members of the MMP family have been described in humans, which are classified into subfamilies according to their structure, substrate specificity, and proteolytic function, including collagenases, gelatinases, stromelysins, matrilysins, metalloelastase, enamelysin, membrane metalloproteases (MT-MMPs), and other MMPs [11,12,13]. The activity of the MMPs is regulated physiologically in a meticulous way to avoid the interruption of the architecture of the tissue [14,15]; however, this activity seems to be uncontrolled in cancer, since different studies have shown increased levels of several MMPs in various cancerous tissues, including breast tumors [16,17]. Some studies have also shown significant associations between tumor aggressiveness and elevated MMP expression. For example, distant metastases from BC have been correlated with high levels of multiple MMPs, including MMP-1, MMP-2, MMP-7, MMP-9, MMP-11, and MMP-13 [18]. Upregulation of several MMPs has also been associated with poor outcome in BC [19].

It has been shown that some members of the family of MMPs promote tumor growth, angiogenesis, epithelial–mesenchymal transition (EMT), and premetastatic niche formation in cancer patients, as is the case with MMP-1, MMP-2, MMP-3, and MMP-9 [20,21,22]. The balance between MMPs and their tissue inhibitors (TIMPs) plays a crucial role in cancer progression and metastasis [23]. Our previous work has shown the involvement of MMP-3, MMP-9, TIMP-3, and TIMP-4 in response to radiotherapy in breast cancer patients, suggesting their utility as potential prognostic and predictive biomarkers for this pathology [24]. In breast cancer, several strategies for the development of inhibitors with therapeutic potential targeting the MMPs were discussed by Radisky et al. [11,19].

MMP-1, also known as collagenase-1 or interstitial collagenase, has a gene locus on chromosome 11q22.3, i.e., MMP-1 is coded on the q arm of chromosome 11. MMP-1 is part of the family of collagenases and is able to degrade interstitial collagens I, II, and III, resulting in denatured collagen or gelatin, and its upregulated expression status has been detected among several kinds of malignant tumors [25,26,27]. As with many other MMPs, the levels of MMP-1 are very low in most cells under physiological conditions but are upregulated in inflammatory conditions and autoimmune disease. MMP-1 is synthesized by normal cells such as macrophages, fibroblasts, and dendritic cells, and in turn is responsible for promoting cell growth. It is the only MMP capable of degrading all types of collagen in the mammary gland and plays a key role in the degradation of stromal fibers in several diseases, including BC [28].

MMP-2 and MMP-9, also called gelatinases A and B, respectively, direct their proteolytic activity to the degradation of denatured interstitial collagen or gelatin, as well as to collagen types IV and V of the basement membrane. MMP-2 has a gene locus on chromosome 16q13-q21 and is physiologically expressed by the stromal cells of most tissues. MMP-9 is also a type IV collagenase that has a gene locus on chromosome 20q11.2-q13.1. MMP-9 is produced by a variety of cells, including epithelial cells, fibroblasts, keratinocytes, osteoblasts, dendritic cells, macrophages, granulocytes, and T-cells [29]; however, its expression can be induced in cases of tissue remodeling, such as embryonic development, scarring, or growth, as well as tumor invasion [30,31]. Stimulation of angiogenesis is an additional role assigned to MMP-9, through its control over the availability of vascular endothelial growth factor (VEGF), which is essential for tumor neovascularization. MMP-9 is synthesized by inflammatory cells, which stimulate angiogenesis by releasing sequestered VEGF, allowing its interaction with VEGF receptors [32]. 

It is known that metastatic BC cells prefer certain organs to establish secondary tumors, such as bone or lung [33,34]; therefore, the dissemination of cancer cells is not a random mechanism, but rather it seems to require the formation of a receptive environment, the so-called premetastatic niche. MMP-2 also participates in this important step of the carcinogenesis process [35].

MMP-3, also known as stromelysin-1, has a gene locus on chromosome 11q22.3. Structurally, MMP-3 possesses some unique characteristics and participates in the breakdown of the adherent junctions mediated by E-cadherin, which means that the tumor cells lose contact with the surrounding cells, promoting the invasion capacity of the tumor cells. This MMP promotes the epithelial–mesenchymal transition, a process associated with structural and functional changes in the epithelial cells that allow their migration through the basement membrane [36,37]. MMP-3 degrades collagen types II, IV, and IX, as well as a variety of proteoglycans, elastin, fibronectin, and laminin. MMP-3 may also activate other MMPs necessary for tissue remodeling including, MMP-1, MMP-7, and MMP-9. MMP-3 has been detected in the nuclei of cultured chondrocytic cells and in normal and osteoarthritic chondrocytes in vivo, as well as in the nuclei of hepatocytes.

Studies carried out on human breast cancers have reported that the stromal fibroblasts that surrounded the tumor cells, not the tumor cells themselves, are responsible for producing the stromelysins [38].

We hypothesized that the MMP expression pattern could be a potential biomarker of BC diagnosis; thus, the aim of this retrospective cohort study was to analyze the immunohistochemical expression of MMP 1, MMP-2, MMP-3, and MMP-9 in tumoral and non-tumoral breast tissues to identify potential tumor markers for BC. The results suggest that MMP-1 and MMP-3 might be associated with BC development, highlighting the need for further functional analysis of their role in breast cancer.

## 2. Results

### 2.1. Clinicopathological Features of Patients

Clinical and pathological data for the studied BC cases are presented in Table 1. A total of 154 women were included in the case group, while 42 women were included in the control group. Clinical and epidemiological characteristics were only investigated for the case group. Descriptive analysis of the study population (cases) showed that the mean age of the women in the study was 63.34 ± 15.30 years; the dates in which BC was diagnosed were between 2015 and 2020, with a clear predominance of BC prevalence in 2017, with a total of 54 patients or 35.1%. Of the 154 patients with cancer, it was found that 53 women had some form of associated risk factor, among which tobacco and obesity showed high percentages of 35.8% and 30.2%, respectively, while 3.8% of patients had a family history of cancer. Of the 100% of the cases, 69.3% the tumors were located in the left breast with involvement of several quadrants. The most prevalent histological type of mammary carcinoma was infiltrating ductal carcinoma (CDI) at 87.7%. It was also observed that 54.2% of our patients had lymph node involvement and 73.5% had positive hormone receptors (estrogen and progestogens). The levels of tumor extension were classified using the TNM system, which in turn were grouped into tumor stages for prognostic evaluation. In total, 46% of the women were classified as stage II (EIIA-EIIB) and only 4.4% were classified as stage IV. Most patients with BC received a combination of surgery (CX) with chemotherapy (QT) or radiotherapy (RT) as their medical treatment, while 27.3% were treated only with CX; of these women who were diagnosed and treated, 10.1% suffered breast tumor recurrence. It was also observed that of the total number of cases, 32% suffered another type of cancer (ovary, endometrium, renal, colon, skin, or stomach); the most common site of tumor invasion was bone tissue at 20%, followed by the lungs at 16.0%. Analyzing the total number of the cases, a global survival rate of 95.6% was found; that is, 131 patients were alive at the end date of the study, with a follow-up patient mortality rate of 4.4% in 2020.

### 2.2. Immunohistochemical Expression of MMPs

Of the studied population of women (controls-cases), not all of them presented immunohistochemical expression of MMP-1-2-3-9 (percentage-intensity) in the mammary tissue analyzed with and without tumor disease. The study variables expression of MMP-1 intensity and MMP-3 percentage and intensity (Table 2) showed statistically significant associations with the variable group (control–case, *p* = 0.043 Chi-square, *p* = 0.018/0.025 Chi-square, respectively). No statistically significant associations were found (*p* ≥ 0.05) for MMP-2 and MMP-9 with the variable group (cases-controls) (Table 2).

**Figure 1:**
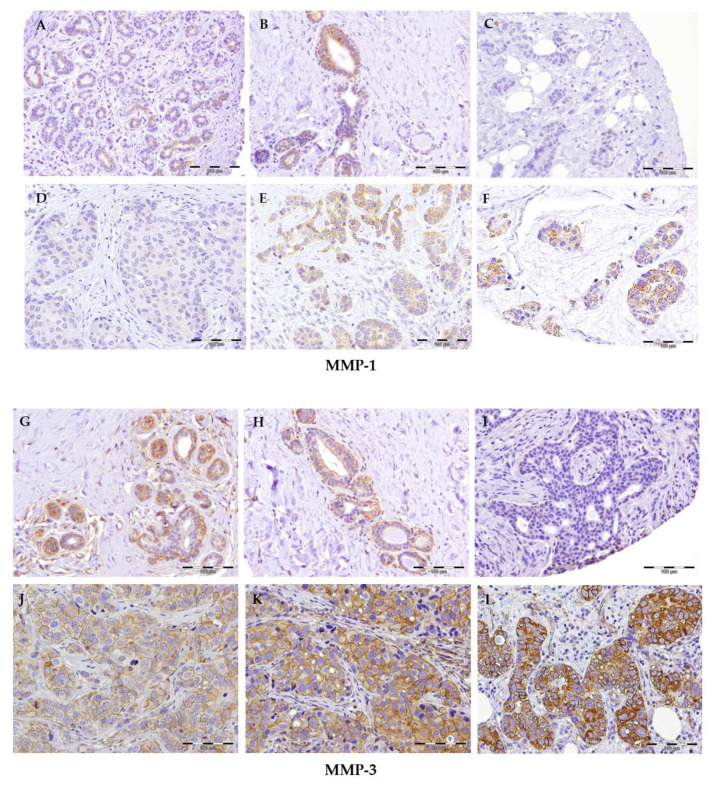
Immunostaining of MMP-1 (ABCAM, (**A**–**F**)): (**A**,**B**) sections of normal breast tissue; (**C**–**F**), sections of breast carcinoma with different level of staining intensity; (**C**) absence of staining; (**D**) weak staining; (**E**) moderate staining; (**F**) intense staining. Immunostaining of MMP-3 (AGENT, (**G**–**L**)): (**G**,**H**) sections of normal breast tissue; (**I**–**L**) sections of breast carcinoma with different levels of staining intensity; (**I**) absence of staining; (**J**) weak staining; (**K**) moderate staining; (**L**) intense staining. The length of the scale bar is 100 μm.

Figure 1 shows the immunostaining of MMP-1 and MMP-3 in the sections of the mammary tissue studied. Staining with anti-MMP-1 was only observed in the cytoplasm of some glandular cells in normal tissue specimens (Figure 1A,B). Reactivity in the cytoplasm of the tumor cells and occasionally in some cells of the stroma was found for tumoral tissues. Four different levels of staining intensity are described (non-staining, weak, moderate, and intense, Figure 1C–F).

Anti-MMP3 antibody in normal breast tissue shows reactivity in the cytoplasm of glandular cells, especially myoepithelial cells, with different levels of intensity. Reactivity is also observed in the vascular endothelium cells and in some cells of the inflammatory infiltrate (Figure 1G,H). Staining is also observed in the cytoplasm of tumor cells. Four different levels of staining intensity are described (non-staining, weak, moderate, and intense, Figure 1I–L). In some cells, the cytoplasmic staining results in membrane enhancement.

We found no relationship between overexpression of MMP-1 and MMP-3 with the clinicopathological characteristics of patients included in this study (age, risk factors, tumor stage, lymph node involvement, treatment, tumor invasion). Nevertheless, of the total number of patients analyzed with cancer, we identified that MMP-1 staining intensity and MMP3 staining percentage and intensity were higher in postmenopause patients, in those with positive hormone receptors, and in the histological ductal carcinoma type (Table 3).

## 3. Discussion

This study was performed to analyze the immunohistochemical expression of four human specific MMPs in specimens from normal and BC tissue. This study aimed to establish a panel of MMP expression as possible biomarkers for the diagnosis of breast cancer. The present study shows that there is a statistically significant association between the immunohistochemical expression of MMP-1 and MMP-3 in the breast tissue of women suffering BC and their expression in the tissue of patients without tumor disease. Immunostaining of MMP-1 and MMP-3 was higher in early stages of the disease, suggesting the strongest role of both MMPs being at the beginning of BC development.

BC is the most common malignant tumor among women worldwide [39]. In recent years, considerable progress has been made in the early detection of BC, allowing higher survival and cure rates in patients diagnosed with this disease; therefore, novel prognostic indicators are necessary to further improve the prognosis of breast cancer patients.

The tumor microenvironment (TME) is increasingly recognized as a key player in tumor progression and as a promising therapeutic target in breast cancer [40]. The TME is composed of ECM, as well as several cellular elements and soluble factors evolving a network of proteins and signalling molecules that play important roles in breast cancer progression and metastasis [41]. The breast TME is modulated by the ECM and extracellular vesicles [42]. The cancer microenvironment often differs from healthy tissue via ECM degradation of protein concentrations. Major alterations occur in the ECM as breast cancer progresses. The epigenetic machinery plays a central role in generating an immunosuppressive environment for cancer growth. For this reason, epigenetic alterations affecting immune cell function in the tumor microenvironment represent a growing area of investigation [43].

There are distinct multilayered epigenetic mechanisms that regulate MMPs. DNA methylation of the regulatory genes may indirectly affect the expression of MMPs in malignancy. Falzone et al. [44] have described the intragenic methylation as a mechanism responsible for the *MMP-9* upregulation in cancer. Nevertheless, some results suggest that elevated or ectopic expression rather than MMP gene methylation-driven silencing might link MMPs with tumorigenesis [45]. On the other hand, microRNAs (miRNAs) regulate the expression and function of extracellular matrix molecules and are often dysregulated in BC [46,47]. Particularly, miRNAs have been a focus of interest in the post-transcriptional regulation of MMPs [48]. Different mechanisms in which miRNAs regulate MMPs have been analyzed in several contexts of tumor invasion, EMT, and ECM remodeling by some authors [49]. Moreover, microRNAs can control breast cancer development, invasion, and migration directly and indirectly through regulating specific MMPs. MiRNAs are also involved in the downregulation of *TIMP-1* and *TIMP-3* in breast cancer [49]. Additionally, miRNAs target chromatin-remodeling histone deacetylases (HDACs), leading to altered MMP activity. In healthy states, there is a physiological balance between activation and inhibition of proteolytic degradation by expression of TIMPs and MMPs. In cancer states, this balance seems to be disrupted [49]. MiR-21 plays an important role in breast cancer. In this regard, miR-21 was shown to target *MMP-3* expression to regulate breast cancer invasion [50], and is found in breast malignancy with high proliferation, advanced-stage, and aggressive phenotypes, such as pregnancy-associated breast cancer [51]. MiR-206 is involved in the downregulations of *MMP-2* and *MMP-9* [52]. The upregulation of miR-103/107 was shown to be associated with metastasis and poor outcome of breast cancer patients [53]. The downregulation of miR-210 was reported to be inversely correlated with cancer aggressiveness and metastatic capability [54]. Other studies have shown increases of breast cancer cell invasion and migration as well as metastasis associated with higher *MMP-9* activity caused by miR-182 regulation. Chu et al. showed that overexpression of miR-519d significantly suppressed proliferation, migration, and metastasis of breast cancer cells by targeting MMP-3, suggesting that the novel molecular correlation between miR-519d and *MMP-3* may become a potential therapeutic approach for breast cancer treatment [55].

Extracellular proteinases such as MMP maintain homeostasis of the ECM and are important key players in the tumor microenvironment. MMPs are a subclass of ECM degradation proteins with concentration differences between healthy and cancer tissues. Changes in the ECM and the interactions between cells and the ECM, with a particular focus on MMPs, have been well documented [56]. Additionally, MMP expression alters the rigidity, porosity, and many other characteristics of the ECM, facilitating cell migration and invasion.

MMPs are involved in the multistep processes of EMT and cancer progression; therefore, they have been considered as potential diagnostic and therapeutic biomarkers for several types of cancer [57]. The initiating step for cancer cells to acquire migratory potential is the EMT, which refers to the reprogramming that occurs in genetically and epigenetically modified cells [58]. E-cadherin has been proposed as an EMT indicator and as a direct target for MMP-dependent shedding, suggesting a direct role for MMPs in disassembly of cell junctions [59]. Some authors have described that Wnt1-induced EMT is associated with MMP-3 activation and that this inhibition resulted in repression of EMT characteristics [60]. MMP-3 is responsible for rendering several active proMMPs, and specifically Suzuki and his colleagues reported the transformation of proMMP-1 to the completely active MMP-1 form by MMP-3 [61].

The overexpression of MMP-1 and MMP-3 is associated with the clinicopathological characteristics of several malignancies [62,63]. This study shows no relationships between expression of MMP-1 and MMP-3 and age, histological type, lymph node affectation, treatment, or hormone receptors for BC (Table 3). This is the main limitation of this study. Nevertheless, of the total number of patients analyzed with cancer, we identified that MMP-1 staining intensity and MMP-3 staining percentage and intensity were significantly increased in the cancerous tissues by 62.3% and 67.5%, respectively, compared to the normal mammary tissues. Preclinical studies revealed that overexpression of MMP-1 plays a role in initiating mammary tumorigenesis through breaking down stroma and disseminating growth factors and mitogens for epithelial cells [64]. Abnormal expression of MMP-1 was identified in several types of malignant cancers [65,66], although its expression status and prognostic merit in BC remain unclear. Some studies have found that elevated expression of MMP-1 can promote the local growth and formation of brain metastases by breast cancer cell [67]. High *MMP-1* gene expression has also been reported to predict for a lower overall survival rate in invasive breast carcinoma [68] and poorer prognosis in patients treated with systemic therapy [69,70]; thus, the expression of MMP-1 is a significant prognostic indicator and a potential drug target for BC [70].

The active participation of MMPs in the different stages of tumor progression is based on various clinical observations related to the expression of these enzymes in different types of human metastatic cancers, as well as on the matrix proteins that are modified by them [71]. Our results show higher immunohistochemical expression levels for MMP1 intensity and MMP-3 percentage and intensity for early-stage breast cancer (EI, EII). This fact would support the interplay between these two MMPs and the biological roles of MMPs related to different steps of carcinogenesis.

MMP-3 is highly expressed in the mammary gland, where it functions to regulate branching morphogenesis and postlactational involution [72]. On the other hand, MMP-3 provides an example of an MMP that can be either protective or protumorigenic in relation to growth [73].

Some studies have been published on the roles of MMP-1 in BC progression and metastasis [74,75]. Other authors have shown the important roles of MMP-3 in tumor progression and overall survival [76]; however, unlike our study, none of the other studies have measured the expression of four MMPs in the biopsies of cancer patients. Our results show that only the expression of MMP-1 and MMP-3 in tumor tissue could be related to the progression of BC and suggest prioritizing these MMPs as candidates for development of therapeutic strategies in these patients.

Few studies have evaluated the immunohistochemical expression of MMP-2 and MMP-9 in BC and fibroadenoma. Some authors have found significantly higher MMP-2 and MMP-9 protein expression in BC cells than in fibroadenoma [77]. Sampaio et al. [78] showed significantly higher expression of metallotionein-1, a membrane-type 1 MMP, in BC than in fibroadenoma. A study published by Li et al. involved 270 patients with BC and consecutive negative lymph node cases who received radical mastectomy or modified radical mastectomy, concluding that MMP-2 and MMP-9 are unfavorable prognostic factors in BC patients. They might be potential predictive factors for adjuvant systemic therapy [79]. MMP-2 and MMP-9 are also involved in each stage of breast-cancer-to-bone metastasis [80]. For these reasons, MMP-2 and MMP-9 have been considered as reliable biomarkers for the prediction of BC prognosis [54] and for metastasis development [55,56].

In this study, MMP-2 and MMP-9 staining showed no significant differences between case and control groups, while a role of MMP-2 and MMP-9 as biomarkers for the prediction of BC progression and metastasis was not supported. These findings are not usual given that the expression levels of MMP-2 and MMP-9 in BC have been described in different studies, since not only do they exhibit proteolytic activity against basal membrane proteins, which translates into tumor invasion, but also influence tumor growth, angiogenesis, and premetastatic niche formation [81,82,83,84]. It is important to highlight that uneven expression levels of several MMP have been found in BC. This could be due to differences in the commercial companies supplying the primary antibody and the methods of immunohistochemical staining used. Currently, there is no consensus on the threshold for MMP overexpression as assessed by immunohistochemistry. Additionally, the cut-off values for percentages or staining intensity levels may differ between studies, resulting in inconsistent positivity rates and predictive values for MMP overexpression. This may be an important source of heterogeneity and could limit the clinical use of MMP expression for the diagnosis of BC. It is also important to consider publication bias in the analysis of MMP overexpression, since studies with negative results may tend to be unpublished.

MMPs are involved in many biological processes and could be important biomarkers for cardiovascular disease, musculoskeletal disorders, and cancer. It is important to consider that the activities of MMPs may vary during disease due to differences in the proteolytic activities of MMPs towards different substrates [85,86,87]; thus, targeting MMPs is a complex task given that individual MMPs act in different cancers and at distinct stages of cancer progression. Pursuing only MMPs expressed by the specific tumor would be a new step torward personalized medicine. Several MMPs are strongly implicated as promising targets for breast cancer therapy. Considering that the efficacy of the therapy with MMPIs drastically decreases with the progression of the disease, it can be hypothesized that inhibition of MMPs could be effective in limiting tumor progression during its initial phase [12]. Some studies [11,12,19,88] have analyzed different strategies for development of inhibitors with therapeutic potential that are capable of selectively targeting the MMPs most responsible for tumor promotion, with special consideration of the potential of biologics including antibodies and engineered proteins based on the TIMP scaffold. Napoli et al. [12] showed the involvement of MMP-9 in the degradation of ECM and the consequent progression of melanoma, as well as the potential therapeutic implication of both endogenous and exogenous MMPIs for the design of new therapeutic protocols for melanoma patients. Most of the MMPIs evaluated in clinical trials to date have failed, causing major musculoskeletal toxicity and failing to improve clinical outcomes [89,90]. The reason for this could be that these trials studied patients with stage IV disease. New trials should enrol patients with high-risk disease that is not yet clinically or pathologically metastatic. On the other hand, the drug should be given prior to surgery, in the so called “window of opportunity” between the time of diagnosis and surgical excision, or postoperatively in the adjuvant setting. This would help to identify and validate biomarkers of enzymatic inhibition and metastasis as a proxy for clinical success [88]. In this regard, the D-Care study (NCT01077154) investigated denosumab, a drug with a similar MMPI design, in the neoadjuvant or adjuvant setting for patients with stage II or III breast cancer at high risk of recurrence. This study demonstrated that denosumab improves bone-related outcomes for women with high-risk early breast cancer.

Despite recent advances in our knowledge of MMPs, multiple functional aspects of these proteases remain unknown [58]. Therefore, we believe that more studies are needed to confirm any of the hypotheses proposed due to the lack of evidence in the literature on this subject.

## 4. Materials and Methods

### 4.1. Patients

This was a retrospective cohort study on 196 elderly patients undergoing breast surgery by the General Surgery Services of the Hospitals associated with the Biobank of Granada, in a period from July 2015 to July 2020. The group of cases was composed of 154 women diagnosed with BC and 42 women without tumor disease belonging to the control group (patients undergoing surgery because of benign breast disease such as fibroadenoma). Written informed consent was obtained from all cases and control subjects involved in the study.

The data and samples were managed through the Biobank for Research of the San Cecil-io-Granada University Hospital, belonging to the National Biobank Network (Project RD09/0076/00148), ensuring the integral treatment of the samples and associated data in accordance with Law 14/2007 of July 3 on Biomedical Research. The study was conducted according to the guidelines of the Declaration of Helsinki and was approved by the Provincial Research Ethics Committee of Granada. The biographical, clinical, and anatomopathological information was obtained only for the group of cases, recording the following data: age, date of diagnosis, risk factors, menopause, affected breast, tumor location, histological type, tumor stage, hormonal receptors, medical treatment established, lymph node involvement, tumor recurrence, metastasis to other organs, and mortality at 5 years.

### 4.2. Immunocytochemical Staining

Breast tissue samples were obtained from both groups to be included in several tissue matrices (TMA), facilitating the processing, staining and interpretation, and successive titration of antibodies for subsequent immunohistochemical assessment. Inmunohistochemistry was carried out on 3µm TMA sections, fixed in 10% buffered formalin, and embedded in paraffin using both PTLink and AutostainerLink (Dako, Glostrup, Denmark). Antibodies for MMPs were obtained from ABCAM (MMP-1, -2, -9) and ABGENT (MMP-3).

The staining process was carried out simultaneously in all sections stained with the same antibody. Both positive and negative controls (replacing the primary antibody with PBS) were made for each antibody used. If there were published reactivity levels for each of the used antibodies, different tissues were considered as positive controls. Assessment of the staining of MMPs was independently evaluated by two pathologists who were blinded to the patients’ clinicopathologic data. Disagreements were resolved by discussion in a meeting to obtain the results. The staining stratification was established based on two scores: (1) the proportion score representing the fraction of positively stained cells (≤50%–>50%, respectively); (2) the intensity of the staining (weak, moderate, or intense). This assessment allows for a semiquantitative estimate of the expression levels of protein in the tissue section. The two scores were added and the final definition of every section was obtained.

### 4.3. Statistical Analysis

The statistical program IBM-SPSS V.26.0 (SPSS Inc., Chicago, IL, USA) was used for the statistical analysis. The Shapiro–Wilk test was applied to verify the normality of the quantitative variable (age at diagnosis of the disease). Further, the age variable was categorized by age range (≤50 years, 51–70 years, and >70 years). The results for the categorical variables are expressed in percentages and the quantitative variables are expressed as means ± standard deviations, minimum and maximum values, and 95% CI values. For the bivariate analysis, Chi-square test was used to compare proportions between groups. Fisher’s exact test was used (Table 2) when the validity conditions were not met.

## 5. Conclusions

The results of this study suggest increased MMP-1 and MMP-3 expression in BC tissue compared to normal breast epithelium tissue. Regarding the association between MMP-1 and MMP-3 expression and other clinicopathological prognostic factors, we could not find significant relationships between the expression of these biomarkers and age, histological type, lymph node affectation, treatment, or hormonal receptors. MMP-1 and MMP-3 are involved in the maintenance of the angiogenic phenotype; thus, inhibition of these proteinases may be of value both in preventing breast cancer and in blocking metastasis of established tumors. As such, the use of MMP inhibitors in patients with early-stage cancer should be considered, as it has mainly been limited to patients with advanced disease to date.

## Figures and Tables

**Table 1:** Descriptive analysis of the study population (cases).

Independent Variables		No. of Patients	Percentage
Age interval	≤50 years	44	28.6
	51–70 years	53	34.4
	>70 years	57	37.0
Risk factors	First-degree family history of BC	2	3.8
	Personal history of other tumors	6	11.3
	History of benign breast lesions	4	7.5
	Smoking	19	35.8
	Obesity	16	30.2
	More than one risk factor	6	11.3
Menopause	Premenopause	47	31.1
	Postmenopause	104	68.9
Affected breast	Right breast	39	30.7
	Left breast	88	69.3
Histological type	Infiltrating ductal carcinoma	135	87.7
	Infiltrating lobulillar carcinoma	13	8.4
	Other carcinomas	6	3.9
Lymph node involvement	No	70	45.8
	Yes	83	54.2
Tumor stage	EI:IA-IB	26	23.0
	EII:IIA-IIB	52	46.0
	EIII:IIIA-IIIB-IIIC	30	26.5
	EIV with any TNM	5	4.4
Hormone receptors	Estrogenics and Progestogens +	72	73.5
	Estrogenics and Progestogens −	18	18.4
Treatment	Surgery + Chemotherapy + Radiotherapy	52	33.8
	Surgery + Chemotherapy	49	31.8
	Surgery + Radiotherapy	11	7.1
	Surgery	42	27.3
Tumor recurrence	No	125	89.9
	Yes	14	10.1
Tumor invasion	Bone	5	20.0
	Lung	4	16.0
	Brain	2	8.0
	Liver	1	4.0
	Association with another cancer	8	32.0
	Metastasis to more than one organ	5	20.0
Follow-up patientMortality in 2020	Dead	6	4.4
	Alive	131	95.6

**Table 2:** Statistical analysis of interactions between MMP-1, MMP-2, MMP-3, and MMP-9 (percentage and intensity) and the variable groups (case and control).

Study Variables		Group			*p*
		Case	Control	Total	
MMP-1 percentage	≤50%	29	9	38	0.073
	>50%	117	16	133	
MMP-1 intensity	Weak	96	22	118	0.043
	Moderate	53	4	57	
	Intense	5	NA	5	*
MMP-2 percentage	≤50%	126	22	148	0.139
	>50%	24	1	25	
MMP-2 intensity	Weak	137	21	158	0.996
	Moderate	13	2	15	
	Intense	NA	NA	NA	*
MMP-3 percentage	≤50%	57	16	73	0.018
	>50%	90	9	99	
MMP-3 intensity	Weak	104	12	116	0.025
	Moderate	43	13	56	
	Intense	7	NA	7	*
MMP-9 percentage	≤50%	146	28	174	0.421
	>50%	NA	NA	NA	*
MMP-9 intensity	Weak	146	28	174	0.326
	Moderate	NA	NA	NA	*
	Intense	NA	NA	NA	*

* Statistical analysis could not be performed with the intense category for MMP-1 and MMP-3 because immunostaining in breast tissue sections was only found in cases and not in controls. The intense category was not found in either normal or tumor breast biopsies for MMP-2 and MMP-9.

**Table 3:** Statistical analysis between variables MMP-1 and MMP-3 expression and clinical–pathological variables.

		MMP-1 Intensity			MMP-3 Intensity			MMP-3 Percentage		
		Weak	Moderate	*p*	Weak	Moderate	*p*	≤50%	>50%	*p*
Age interval	≤50 years	32	10	0.168	35	8	0.177	12	31	0.210
	51–70 years	31	20		31	17		20	28	
	>70 years	33	23		38	18		25	31	
Tumor stage	EI	19	7	0.549	19	5	0.766	10	14	0.978
	EII	33	16		36	14		21	29	
	EIII	19	10		18	9		10	17	
	EIV	2	3		4	1		2	3	
Menopause	Premenopause	34	11	0.064	37	9	0.115	14	32	0.204
	Postmenopause	60	41		65	33		41	57	
Lymph node involvement	No	42	25	0.730	48	19	0.856	26	41	0.510
	Yes	54	27		55	24		30	49	
Tumorrecurrence	No	79	42	1.000	87	35/2	0.510	47	75	1.000
	Yes	9	5		10	2		5	7	
Affected breast	Right breast	29	8	0.063	23	13	0.520	10	26	0.307
	Left breast	51	34		62	25		33	54	
Hormone receptors	Estrogenics and Progestogens +	41	29	0.394	45	26	0.085	22	49	0.348
	Estrogenics and Progestogens −	13	4		14	2		8	8	
	Estrogenics +	5	3		7	1		3	5	
Risk factors	First-degree family history of BC	1	1	0.916	1	1	0.056	1	1	0.151
	Personal history of other tumors	4	2		1	5		1	5	
	History of benign breast lesions	3	1		3	1		1	3	
	Smoking	10	8		12	6		3	15	
	Obesity	10	6		14	2		9	7	
	More than one risk factor	4	1		3	3		1	5	
Histological type	Infiltrating ductal carcinoma	84	47	0.748	91	38	0.272	48	81	0.502
	Infiltratinglobulillarcarcinoma	8	5		8	5		6	7	
	Othercarcinomas	4	1		5	0		3	2	
Treatment	Surgery +Chemotherapy+ Radiotherapy	31	18	0.984	36	15	0.856	18	33	0.863
	Surgery +Chemotherapy	32	16		31	13		17	27	
	Surgery +Radiotherapy	7	4		9	2		4	7	
	Surgery	26	15		28	13		18	23	
Tumorinvasion	Bone	4	1	0.674	4	0	0.216	2	2	0.912
	Lung	3	1		3	1		1	3	
	Brain	1	1		2	0		1	1	
	Liver	0	1		1	0		0	1	
	Association with another cancer	6	2		3	5		3	5	
	Metastasis to more than one organ	3	2		3	1		1	3	

## Data Availability

The data presented in this study are available on request from the corresponding author.
